# Revealing within-species diversity in uncultured human gut bacteria with single-cell long-read sequencing

**DOI:** 10.3389/fmicb.2023.1133917

**Published:** 2023-02-24

**Authors:** Masato Kogawa, Yohei Nishikawa, Tatsuya Saeki, Takuya Yoda, Koji Arikawa, Haruko Takeyama, Masahito Hosokawa

**Affiliations:** ^1^Research Organization for Nano and Life Innovation, Waseda University, Tokyo, Japan; ^2^Computational Bio Big-Data Open Innovation Laboratory, National Institute of Advanced Industrial Science and Technology, Tokyo, Japan; ^3^bitBiome, Inc., Tokyo, Japan; ^4^Department of Life Science and Medical Bioscience, Waseda University, Tokyo, Japan; ^5^Institute for Advanced Research of Biosystem Dynamics, Waseda Research Institute for Science and Engineering, Tokyo, Japan

**Keywords:** gut, microbiome, single-cell genomics, long-read sequencing, genome

## Abstract

Obtaining complete and accurate bacterial genomes is vital for studying the characteristics of uncultured bacteria. Single-cell genomics is a promising approach for the culture-independent recovery of bacterial genomes from individual cells. However, single-amplified genomes (SAGs) often have fragmented and incomplete sequences due to chimeric and biased sequences introduced during the genome amplification process. To address this, we developed a single-cell amplified genome long-read assembly (scALA) workflow to construct complete circular SAGs (cSAGs) from long-read single-cell sequencing data of uncultured bacteria. We used the SAG-gel platform, which is both cost-effective and high-throughput, to obtain hundreds of short-read and long-read sequencing data for specific bacterial strains. The scALA workflow generated cSAGs by repeated *in silico* processing for sequence bias reduction and contig assembly. From 12 human fecal samples, including two cohabitant groups, scALA generated 16 cSAGs of three specifically targeted bacterial species: *Anaerostipes hadrus*, *Agathobacter rectalis*, and *Ruminococcus gnavus*. We discovered strain-specific structural variations shared among cohabiting hosts, while all cSAGs of the same species showed high homology in aligned genomic regions. *A. hadrus* cSAGs exhibited 10 kbp-long phage insertions, various saccharide metabolic capabilities, and different CRISPR-Cas systems in each strain. The sequence similarity of *A. hadrus* genomes did not necessarily correspond with orthologous functional genes, while host geographical regionality seemed to be highly related to gene possession. scALA allowed us to obtain closed circular genomes of specifically targeted bacteria from human microbiota samples, leading to an understanding of within-species diversities, including structural variations and linking mobile genetic elements, such as phages, to hosts. These analyses provide insight into microbial evolution, the adaptation of the community to environmental changes, and interactions with hosts. cSAGs constructed using this method can expand bacterial genome databases and our understanding of within-species diversities in uncultured bacteria.

## 1. Introduction

The gut microbiota plays a crucial role in regulating host physiology and metabolism, and culture-independent analysis of bacterial genomes has been key to understanding this relationship. However, an estimated half of human gut microbiota species do not have reference genomes, which limits our ability to accurately assign functions to specific organisms and classify the microbiota taxonomically. In recent years, short-read sequencing with assembly and binning algorithms has resulted in a large number of metagenomic assembled genomes (MAGs; Nayfach et al., 2019; Pasolli et al., 2019; Almeida et al., 2021), but these have low assembly quality, including unlinked loci, missing rRNA genes, and chimeric sequences (Bowers et al., 2017; Shaiber and Eren, 2019; Van Rossum et al., 2020). This genome incompleteness has raised concerns about the quality of MAG-derived reference databases and the validity of MAG-based studies, particularly in terms of characterizing intra-species diversity in gut bacteria.

Single-cell genomics is an alternative approach that can recover bacterial genomes in a culture-independent manner by sequencing individual cells rather than populations (Woyke et al., 2017). During this process, a single bacterial cell is isolated, lysed, and the genome is amplified using a technique called multiple displacement amplification (MDA; Lasken, 2007). While MDA generates sufficient DNA with high fidelity and large fragment sizes, it can also introduce chimeric artifacts within a single genome and result in biased coverage of certain genomic regions (Kogawa et al., 2018). As a result, single-amplified genomes (SAGs) often have fragmented and incomplete sequences with errors, and only a minimal number of high-quality draft SAGs and circular SAGs (cSAGs) have been recovered according to the Minimum Information about SAG standards (Rinke et al., 2014; Bowers et al., 2017).

In this study, we used high-throughput single-cell genome sequencing to recover complete genomes from uncultured human gut microbiota. We combined the SAG-gel (SAGs in gel beads sequencing) platform, a massively parallel single bacterial genome sequencing technique (Chijiiwa et al., 2020; Arikawa et al., 2021; Hosokawa et al., 2022; Ide et al., 2022; Nishikawa et al., 2022), with a nanopore long-read sequencer (Bertrand et al., 2019; Moss et al., 2020). We developed a genome assembly pipeline specifically for single-cell long-read sequences and validated its performance using *Escherichia coli* single-cell MDA products. We then applied this pipeline to the three bacterial species in the Clostridiales order, which were obtained from single-cell MDA products of uncultured human gut bacteria. This allowed us to characterize structural variation and mobile genetic elements in these complete, closed genomes.

## 2. Results

### 2.1. Evaluation of the conventional long-lead assembly pipeline using *Escherichia coli* single-cell genomes

Short-read sequencing derived from *E. coli* SAGs can mitigate the effects of chimeras and biases through the co-assembly of multiple data, yielding a draft genome of quality comparable to that of an isolate genome (>97% completeness) as a composite short-read SAG (CSR-SAG; Kogawa et al., 2018). However, in terms of assembly based on short-read sequence information, the contigs obtained from both single cells and isolates are fragmented, numbering around 170. To obtain a circular genome by *de novo* assembly, it is necessary to increase the fragment length of the sequencing reads and extend the length of the contigs to be assembled, necessitating the utilization of long-read sequencing.

Initially, we evaluated long-read SAGs (LR-SAGs) constructed from *E. coli* single-cell MDA products using existing long-read assemblers to identify challenges in the *de novo* assembly of single-cell long-read sequences (scLRs; Supplementary Table S1). First, 96 single-cell MDA products of *E. coli* K-12 were prepared using the SAG-gel platform (Chijiiwa et al., 2020), and five amplified genomes were then randomly selected and individually sequenced using a nanopore sequencer. The scLRs (300 Mb each) were merged into a single file and assembled using three different long-read assemblers: Flye (Kolmogorov et al., 2019), miniasm (Li, 2016), and Canu (Koren et al., 2017; Supplementary Table S1). The LR-SAG produced by Flye, which is renowned for its reliability in sequencing genomes extracted from cultured bacteria, had genome completeness of only 1.53% and lost most of the sequence information. Similarly, the LR-SAG produced by miniasm had genome completeness of 1.17%. In contrast, the LR-SAG produced by Canu, which features a built-in unit for processing chimeric sequences, had high genome completeness of 87.5%. However, this assembly contained 61 contigs, including numerous fragments with the same sequence information, as indicated by a duplication ratio of 1.039.

To enhance the quality of the assembly, we first eliminated chimeras from the scLRs using Canu, and then reassembled them using Flye and miniasm. This resulted in a reduction in the number of contigs (39 and 47, respectively), while maintaining genome completeness of more than 70%. However, both of these assemblies still had a maximum genome completeness of 87.5%, indicating that substantial genomic sequence information was still lost compared to short-read sequencing. An examination of the scLR sequence regions with small relative depth in the LR-SAGs revealed missing sequences from the assembled contigs (Supplementary Figure S1), leading us to posit that improving sequencing biases in the scLRs could augment the assembly quality.

### 2.2. Enhancing genome completeness by reducing bias in single-cell long-read sequences

**Figure 1:**
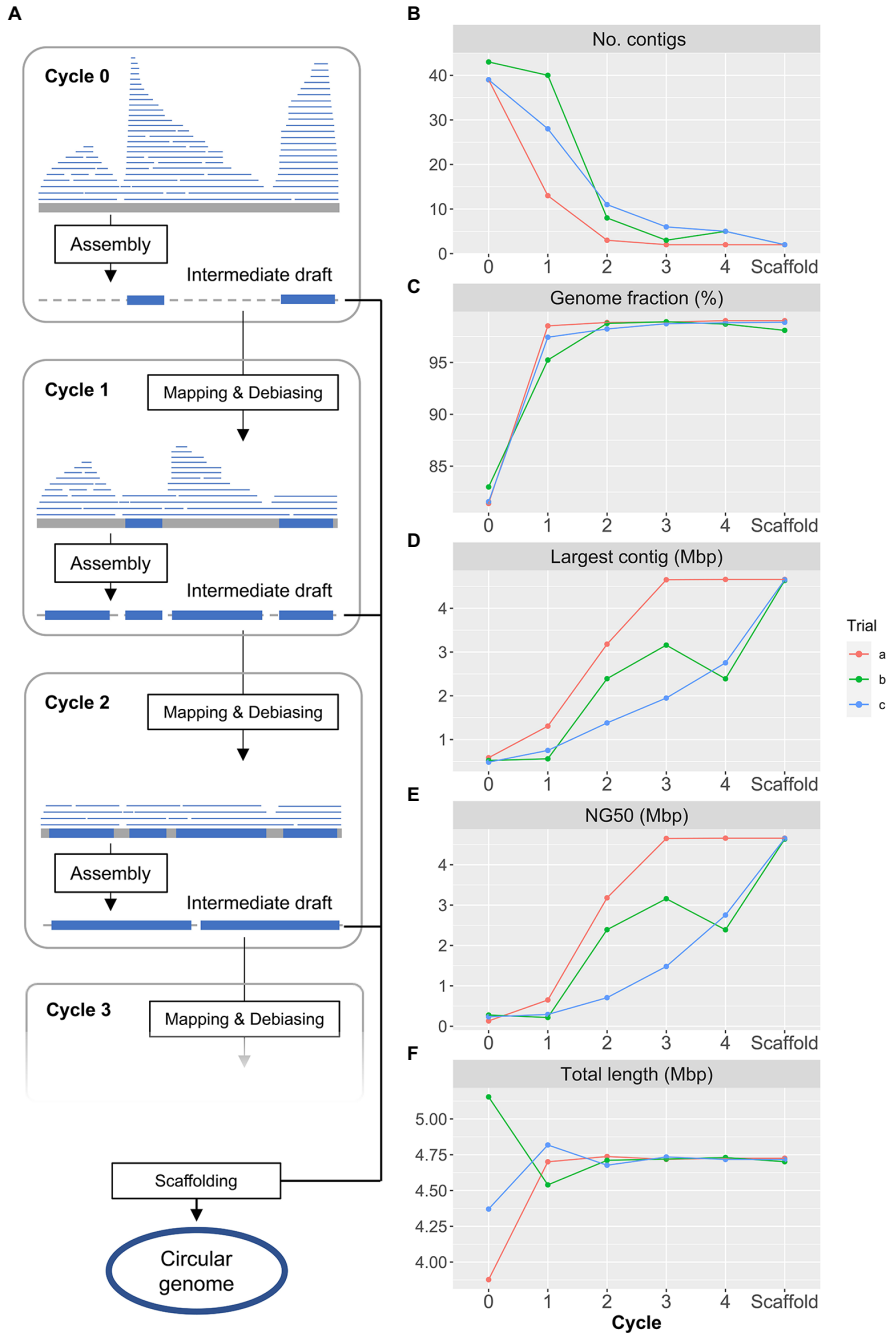
Workflow of single-amplified genome long-read assembly (scALA) pipeline to obtain circular bacterial genomes. **(A)** The assembly of original single-cell long-read sequences (scLRs) obtained from SAGs produces a low-completeness draft genome (LR-SAG) owing to biased sequence depth caused by uneven genome amplification (Cycle 0). To improve the quality of LR-SAG, the assembly of debiased long reads is repeated by read mapping to the pre-assembled intermediate draft LR-SAG (Cycle 1, Cycle 2, …). Finally, circular LR-SAG (cSAG) is obtained by scaffolding multiple intermediate draft LR-SAGs in each cycle. **(B–F)** Quality of LR-SAGs in scALA process (**B**: number of contigs, **C**: genome fraction, **D**: length of the largest contig, **E**: NG50, **F**: total length). The line plot color indicates each scALA analysis trial using the same *E. coli* scLR dataset.

We developed the single-cell amplified genome long-read assembly (scALA) pipeline to assemble draft bacterial genomes from scLRs with amplification bias and chimeric sequences (Figure 1A). This pipeline first removes low-quality reads from the input reads and then constructs reference contigs for the sequence debiasing process. After chimera removal using Canu, the input reads are assembled to the intermediate reference contigs using Flye. The input reads are then mapped against the reference contigs to identify areas with a high read depth, and excess reads are removed to debias the input reads (Figure 1A). The debiased LR-SAGs, which have improved genome coverage breadth, are then constructed by reassembling the bias-reduced reads and used as renewal reference contigs for further debiasing and reassembly. This process is repeated to provide contiguous sequences with improved genome completeness. The consensus sequences are then constructed from the intermediate references obtained during the debiasing and assembly cycle. The final complete genome is obtained by polishing the consensus sequences with single-cell short-read sequences (scSRs) obtained from the same single-cell MDA products.

To evaluate the impact of bias reduction on the completeness of LR-SAGs, we used merged *E. coli* scLRs, which were also used to assess the conventional long-read assembly tools in the previous section (Figures 1B–F, trial-a). After *de novo* assembly, an LR-SAG comprising 39 contigs was obtained from scLRs before debiasing, with a genome completeness of 81.7%. In contrast, an LR-SAG comprising 13 contigs was constructed from scLRs after the first debiasing, with a genome completeness of 99.3%, indicating that bias reduction improved genome completeness. The number of contigs was further reduced to two after repeated debiasing, and a full-length *E. coli* genome sequence with a maximum contig length of 4.66 Mbp was finally obtained. The other contig was the F plasmid sequence from *E. coli*. These results show that repeated debiasing processes can construct a complete SAG from scLRs by increasing completeness and filling sequence gaps, resulting in contig reduction.

The key to obtaining circular SAGs with high reproducibility is the repeated debiasing cycles and the scaffolding of multiple reference contigs produced in the cycles. We observed differences in the number and size of contigs in output genomes for each trial (Figures 1B–F). Three validation trials (a, b, and c) using the same *E. coli* scLRs yielded similar LR-SAGs containing 39–43 contigs before the debiasing cycle, with genome completeness of 81.7–83.2%. Although the debiasing cycles improved genome completeness and contiguity gradually (Figures 1B,C), only one of the three trials resulted in constructing a cSAG after four debiasing and assembly cycles (Supplementary Table S2). This difference is likely due to the use of random values in some parts of the Flye assembly algorithm. While random seeds are typically set for genome assembly to achieve reproducible results, this validation suggests that unintentional random seed setting may prevent the acquisition of cSAGs. The alignment of LR-SAGs (intermediate reference contigs) obtained at each step showed that sequence fragment ends were located at different genomic positions, and BLAST homology searches for each LR-SAG showed that the sequence fragments could be stitched together and extended as the consensus sequence (Supplementary Figure S2). Therefore, we implemented a process in the pipeline to generate a consensus contig as the final output by scaffolding all reference contigs obtained in different assembly cycles. This resulted in the construction of cSAGs from all trials (Figure 1B; Supplementary Table S2).

### 2.3. Genome comparison analysis of gut bacterial single-amplified genomes obtained from different hosts

**Figure 2:**
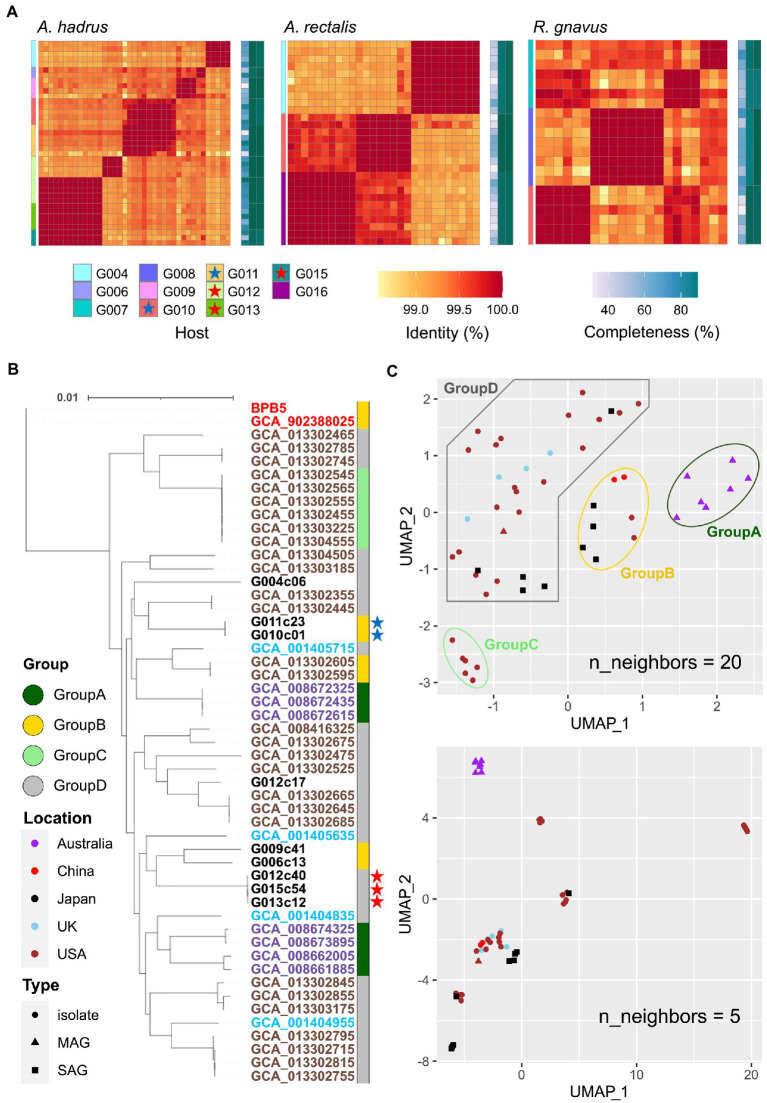
Phylogenetic analysis of gut bacterial SAGs for *A. hadrus*, *A. rectalis*, and *R. gnavus*. **(A)** Heatmaps of average nucleotide identity (ANI) among SAGs of the three species. The tiles on the left indicate the host of obtained SAGs. Colored stars in the host legends indicate cohabitants. Central tiles indicate the ANI between short-read (SR)-SAGs. Tiles on the right represent the completeness of SAGs processed in different steps [left column: raw SR-SAG, middle column: composite short-read SAG (CSR-SAG), right column: composite long-read SAG (LR-SAG)]. As shown in panels **B**,**C**, publicly available *A. hadrus* genomes obtained from isolates or metagenome-assembled genomes (MAG) were compared with our *A. hadrus* SAGs. **(B)** A SNP-based tree of *A. hadrus* genomes based on single nucleotide polymorphisms (SNPs) of the core gene. The label color indicates the country where the *A. hadrus* genome was obtained. The color strip on the right represents the genome group based on Uniform Manifold Approximation and Projection (UMAP) analysis **(C)**. The colored stars are closely related SAGs obtained from the cohabitants. **(C)** UMAP plot of *A. hadrus* genomes based on the presence of homologous gene groups. The point color indicates the country where the bacterial sample was obtained, and the point shape indicates the data type of genome sequences.

We obtained cSAGs of bacterial species obtained from 12 human fecal samples, including two cohabitant groups listed in Supplementary Table S3. First, 96 short-read (SR)-SAGs of human gut bacteria were randomly obtained from single-cell MDA products prepared with SAG gel platform (Chijiiwa et al., 2020), and scSR data were then obtained using the HiSeq sequencing system. After *de novo* assembly of scSR, CSR-SAGs were constructed by integrating SR-SAGs of the same strain (Kogawa et al., 2018). Subsequently, we identified certain bacterial species whose CSR-SAG showed >90% completeness from multiple specimens. As shown in Figure 2A, we targeted three bacterial species, *Anaerostipes hadrus*, *Agathobacter rectalis*, and *Ruminococcus gnavus,* which were shared among hosts and were known as host-health-associated bacteria (Henke et al., 2019; Zeevi et al., 2019; Wang et al., 2021). Most CSR-SAGs of the selected species were qualified as high quality (HQ) or medium quality (MQ) and had high genome completeness (> 95%), comprising over 100 contigs (Supplementary Table S4).

SR-SAG pairwise average nucleotide identity (ANI) clusters suggested the presence of *A. hadrus* strains shared among different hosts, the strain G010c01 and G011c23 or the strain G012c40, G013c12, and G015c54 (Figure 2A; Supplementary Figure S3). Host groups harboring these specific strains were cohabitants. Further, we performed phylogenetic analysis within the species using *A. hadrus* comparative genome set which consisted of nine obtained CSR-SAGs and 43 *A. hadrus* draft genomes from the National Center for Biotechnology Information (NCBI) genome database. We analyzed phylogenies of these strains based on single nucleotide polymorphisms (SNPs) of the common core gene of all genomes in the dataset (Figure 2B). The results suggested that the strain groups with >99.5% ANI from cohabitants were considered shared *A. hadrus* strains between couples or parents and children.

We clustered each strain genome based on the presence or absence of homologous functional genes to compare genomes from different countries. Furthermore, we used Uniform Manifold Approximation and Projection (UMAP) clustering based on Jaccard distances between genomes to classify 52 genomes into four groups (Figure 2C, n_neighbors = 20). The *A. hadrus* genome obtained in Australia showed regionally specific clusters in UMAP analysis (Figure 2C); however, Australian strains were distributed across multiple clades in the SNP-based tree based on core gene sequences (Figure 2B). *A. hadrus* strains G006c13, G009c41, G010c01, and G011c23, whose genomes were obtained in this research, were also located in the same UMAP cluster but different clades. Phylogenetic analysis based on core gene sequence identity did not necessarily represent the functional similarity of the bacteria and acquiring genomic data of geographical strains was essential to predict bacterial traits more accurately. Moreover, UMAP plots reflecting additional local structural variation (Figure 2C, n_neighbors = 5) placed the strains G006c13, G009c41, G010c01, and G011c23 at distant positions, suggesting that obtaining strain-resolved genomes from each host is necessary to understand these differences.

### 2.4. Acquisition of circular single-amplified genomes from human gut bacteria using single-cell amplified genome long-read assembly

We collected 2–8 single-cell MDA products for *A. hadrus*, *A. rectalis*, and *R. gnavus* from each 96-well plate. The pooled MDA products were individually sequenced using nanopore sequencers. The strain-specific LR-SAGs were individually assembled using scALA under optimized conditions. For nine *A. hadrus* strains, LR-SAGs with one-digit contig numbers were obtained, and for two *A. hadrus* strains, a single closed genome sequence of 3.12 Mbp (*A. hadrus* G009c41) and 3.30 Mbp (*A. hadrus* G011c23) was constructed without manual scaffolding (Supplementary Table S5).

To evaluate the assembly accuracy of *A. hadrus* LR-SAG constructed from single-cell MDA products containing chimeric sequences, we tested the alignment of LR-SAGs against the known complete genome of *A. hadrus* strain BPB5. The alignment results demonstrated that only the strain G012c17 LR-SAG was homologous to the strain BPB5 genome (Supplementary Figure S4). The other strains had a large inversion in the 1.7–2.2 Mb region of the BPB5 genome; however, the possibility of misassembly was exceptionally low because this structural variation was common to all eight LR-SAGs. Massive inversions of over 500 kbp are challenging to detect in SR-SAGs because they mainly consist of sequence fragments of 10 of kbp or less. Finally, closed cSAGs of each strain (nine, four, and three genomes for *A. hadrus*, *R. gnavus*, and *A. rectalis*, respectively) were constructed by polishing and gap-filling between contigs of LR-SAG with scSR of the same samples and were used for subsequent analyses (Table 1).

**Table 1:** Circular single-amplified genomes (cSAGs) of *A. hadrus*, *A. rectalis*, and *R. gnavus.*

**Species**	**Strain**	**Genome size (bp)**	**GC (%)**	**Completeness (%)**	**Redundancy (%)**	**tRNA types**	**16S rRNA**	**CDS**
*Anaerostipes hadrus*	G004c06	3,251,112	36.79	95.64	2.52	19	6	3,112
*Anaerostipes hadrus*	G006c13	2,988,610	36.99	97.65	1.34	20	7	2,987
*Anaerostipes hadrus*	G009c41	3,098,900	37.06	98.99	2.68	20	6	2,966
*Anaerostipes hadrus*	G010c01	3,222,401	37.18	95.13	2.01	20	6	3,053
*Anaerostipes hadrus*	G011c23	3,312,423	37.18	99.33	2.01	20	5	3,141
*Anaerostipes hadrus*	G012c40	3,390,800	37.05	97.99	2.01	20	7	3,292
*Anaerostipes hadrus*	G012c17	3,214,912	37.21	99.33	2.68	20	6	3,057
*Anaerostipes hadrus*	G013c12	3,362,201	36.99	98.32	2.68	18	4	3,335
*Anaerostipes hadrus*	G015c54	3,259,135	36.93	94.21	2.01	20	6	3,237
*Ruminococcus gnavus*	G007c17	3,592,770	42.74	97.72	2.44	20	4	3,671
*Ruminococcus gnavus*	G007c21	3,276,103	42.65	90.62	6.14	19	5	3,224
*Ruminococcus gnavus*	G008c02	3,344,851	42.79	99.32	0	20	5	3,198
*Ruminococcus gnavus*	G010c06	3,418,459	43.07	96.37	0	20	4	3,338
*Agathobacter rectalis*	G004c08	2,910,410	41.79	96.98	0	20	5	2,704
*Agathobacter rectalis*	G011c14	3,718,229	41.63	98.55	0	20	5	3,599
*Agathobacter rectalis*	G016c02	3,590,394	41.13	95.89	1.93	20	5	3,362

**Figure 3:**
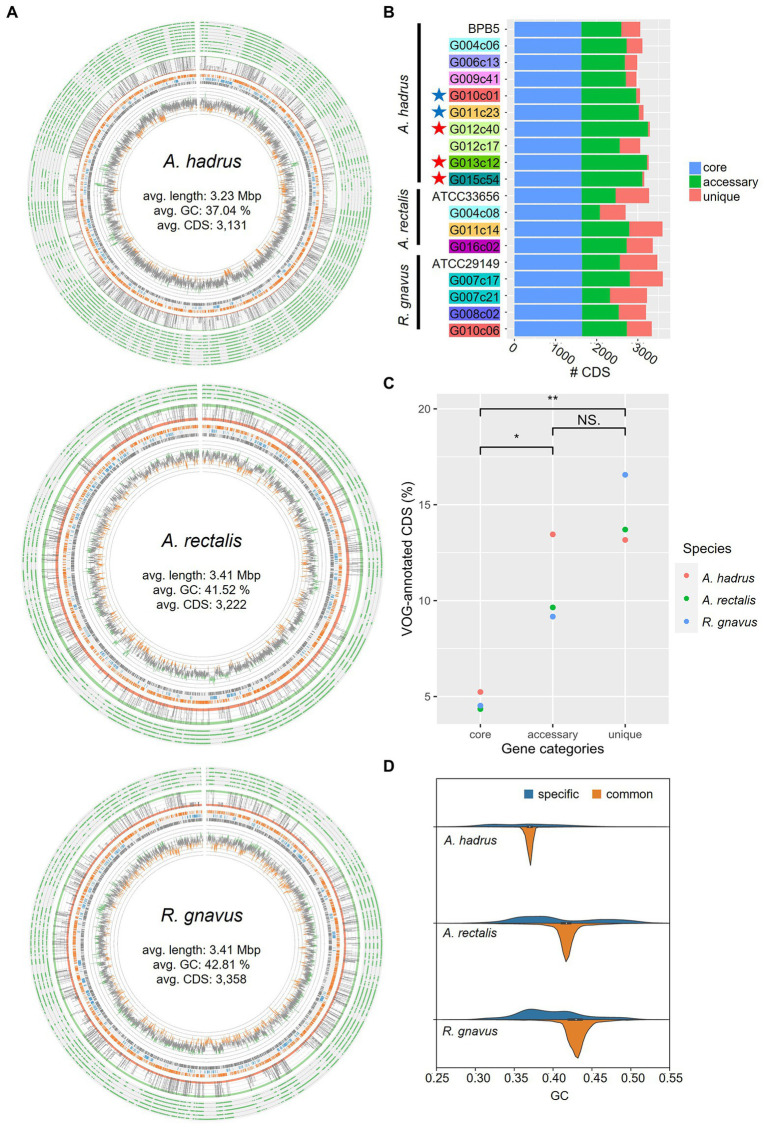
Comparison of the structures of gut bacterial circular single-amplified genomes (cSAGs). **(A)** Genome maps of *A. hadrus*, *A. rectalis*, and *R. gnavus*. Track 1 (outside) tiles indicate the presence (green) or absence of gene clusters in each strain genome. Track 2 is a line plot indicating the number of genomes, including the gene cluster (green ribbon: core gene, orange ribbon: unique gene). Track 3 plots the gene cluster annotations (orange: Kyoto Encyclopedia of Genes and Genomes (KEGG)-annotated clusters, blue: COG-annotated clusters, gray: unannotated clusters). Track 4 is a line plot of GC contents (green: high-GC region, orange: low-GC region). **(B)** Number of homologous gene clusters in cSAGs and reference genomes. The number of coding sequences (CDS) in core orthologous genes (OGs; blue), accessory OGs (green), and unique OGs (red) are shown per cSAG. Highlighted labels indicate the hosts, and colored stars indicate closely related genomes obtained from the cohabitants. **(C)** Accumulation of virus orthologous groups (VOG)-annotated CDS in the strain-specific accessory or unique categories. **(D)** GC content distributions of three bacterial genomes divided into strain-specific and strain-common sequence regions.

Furthermore, we analyzed the closed genome set of three bacterial species, consisting of cSAGs obtained in this study and one known circular genome for each species (CP012098, NC012781, and CP043051). From the results of the pan-genome analysis using the closed genome set, we generated a genome plot of the three species using genome alignment based on homologous gene positions (Figure 3A). Multiple strain-specific sequence regions of up to 100 kbp or more were identified throughout the genome. The pan-genome analysis of each bacterial species revealed that *A. hadrus* strains shared among cohabitants (G010c01-G011c23 and G012c40-G013c12-G015c54) had five-fold fewer unique genes than those of host-specific *A. hadrus* strains (Figure 3B), which highlighted that gut bacteria were shared among cohabitants. Functional annotation of gene sequences obtained from each strain genome using Kyoto Encyclopedia of Genes and Genomes (KEGG; Aramaki et al., 2020) and Virus Orthologous Groups (VOG; Kieft et al., 2020) confirmed that phage-like genes were significantly concentrated in strain-specific sequence regions. The rate of VOG-annotated genes in accessory or unique genes was higher than that in core genes (value of p, accessory vs. core: 0.042, unique vs. core; 0.0081; Figure 3C). Guanine-cytosine (GC) contents of the core genome showed a single peak, whereas GC contents of strain-specific regions containing viral sequences showed a more widely spread distribution with multiple weak peaks (Figure 3D).

### 2.5. Metabolic analysis of strain-specific structural variations observed in circular single-amplified genomes

**Figure 4:**
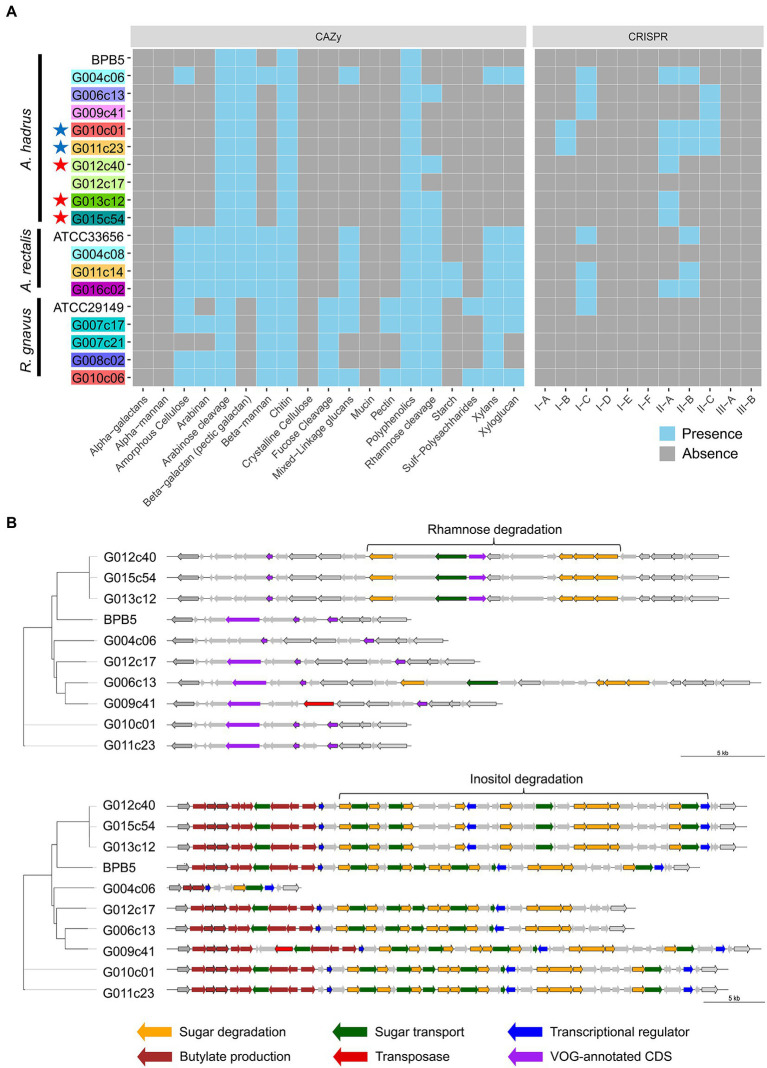
Metabolic analysis of gut bacterial circular single-amplified genomes (cSAGs). **(A)** Heatmap showing various carbohydrate-active enzymes (CAZy) or CRISPR-Cas systems in the genome of each strain. Highlighted labels indicate the host, and colored stars indicate closely related genomes obtained from the cohabitants. **(B)** Structural variation of polysaccharide metabolic pathways in *A. hadrus* genomes. Gene clusters in the same regions are aligned. The left phylogram was inferred from the present pattern of orthogroups in whole genomes.

Metabolic analysis using obtained cSAGs was conducted to examine the manner in which structural variations affect bacterial traits. The screening of functional gene modules showed strain-specific differences in the presence or absence of gene modules associated with CAZy (Carbohydrate-Active enZymes), clustered regularly interspaced short palindromic repeats (CRISPR), and certain functions (Figure 4A). CAZy profile indicated that some strains had the metabolic capacity of more diverse carbohydrates compared with that of other strains of the same bacterial species. For example, amorphous cellulose, beta-mannan, xylans, and xyloglucan metabolic capacities were detected only in the strain G004c06 of *A. hadrus* cSAG set, and only strains G006c13, G012c40, G013c12, and G015c54 showed rhamnose metabolic capacity.

The presence of the gene module related to rhamnose metabolism was visualized using *A. hadrus* cSAGs (Figure 4B). The inositol metabolic system is a gut bacterial metabolic system highlighted in a cohort study using shotgun metagenomic data of the human gut microbiota (Zeevi et al., 2019), suggesting the presence or absence of inositol metabolism gene module in the *A. hadrus* genome was correlated with host body weight and metabolic disease risks. In the above mentioned study, metagenomic reads were mapped to a database of bacterial genomes to detect variable and deletion structural variants based on differences in read depth; all inositol metabolism genes in *A. hadrus* were deleted in approximately 40% of their metagenomic data sets. In contrast, the results of our study showed more diverse structural variations, such as gene rearrangements and partial deletions of functional genes, as well as the presence or absence of the entire inositol metabolic system in *A. hadrus* (Figure 4B). In addition, such as the rhamnose metabolic system analysis, inserted gene sequences could not be detected using mapping-based evaluation owing to the loss in the known *A. hadrus* BPB5 genome.

The number of CRISPR arrays in each genome varied: 2–7, 2–4, and 0–1 loci in the *A. hadrus*, *A. rectalis*, and *R. gnavus* genomes, respectively (Figure 4A). *A. hadrus* genomes were classified into five types based on possession pattern of CRISPR-Cas systems, and transposase genes near the CRISPR-Cas systems indicated that these systems were transferred horizontally. The same types of CRISPR-Cas systems were located on the same regions of *A. hadrus* genomes, suggesting that each CRISPR-Cas system was inherited from the same ancestor strain acquired in the CRISPR-Cas system. Interestingly, CRISPR array sequences exhibited high strain specificity.

### 2.6. Tracing genome structure dynamics of *Anaerostipes hadrus*

**Figure 5:**
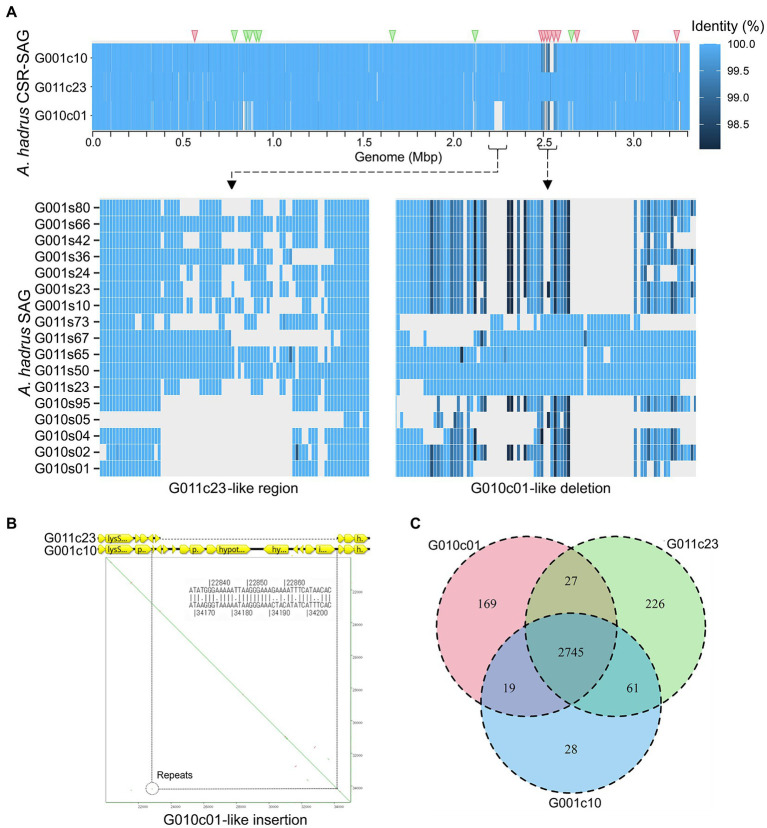
Inter-strain genome structure variation in *A. hadrus* strain SAGs obtained from the same host or a cohabitant at different time points. **(A)** Heatmap showing nucleotide identity of each strain CSR-SAG with the circular single-amplified genomes (cSAGs) of *A. hadrus* G01c23. G011c23, and G001c10 derived from the same host, whereas strain G010c01 was derived from the cohabitant. Triangles at the top of the heatmap indicate SNPs detected upon comparing the three *A. hadrus* strains (red: G001c10-G010c01 common nucleotides, green: G001c10-G011c23 common nucleotides). Detailed view shows raw SR-SAG mapping results, indicating G010c01-like deletion in the G001c10 genome compared with the G011c23 genome. **(B)** Gene map and dot plot of G010c01-like insertion region in the G001c10 genome compared with the G011c23 genome. **(C)** Venn diagram of homologous gene clusters in genomes of the three *A. hadrus* strains.

A total of 9 months after collecting fecal samples containing *A. hadrus* G011c23, a fecal sample was collected again from the same host, and 7 SR-SAGs of *A. hadrus* were obtained using SAG-gel platform. The newly obtained SR-SAGs of *A. hadrus* strain G001c10 demonstrated >99.5% ANI with G011c23 SR-SAGs; therefore, genomic structural variations that occurred over time were investigated. CSR-SAGs constructed from *A. hadrus* G001c10 SR-SAGs were compared with *A. hadrus* G011c23. The G001c10 CSR-SAG had a 50-kbp highly diverse genomic region, including a 20-kbp deletion (Figure 5A) and 12-kbp insertion (Figure 5B), at the position corresponding to 2.3 Mbp of the G011c23 cSAG. Although traces of repeat sequences at both ends of the insertion region were observed, indicating that the region was a prophage sequence (Figure 5B), low homology of repeat sequences suggested that the detected structural variation occurred prior to the last 9 months. This highly diverse region in the strain G001c10 was similar to the sequence structure in the strain G010c01 genome obtained from a close relative of the strain G011c23 host (Figure 5A). Moreover, the sequence structures shared only between G011c23 and G001c10 were identified; thus, the three strains, G010c01, G011c23, and G001c10, were considered distinct. Ortholog (OG) analysis revealed that the strain G001c10 had significantly fewer specific OGs (28) than those of the strain G010c01 or G011c23 (169 and 226, respectively), indicating that the strain G001c10 genome had intermediate genome homology with the strains G010c01 and G011c23 (Figure 5C). Structural differences were also detected in individual SAGs, suggesting that a single strain was dominant in each sample or at each sampling time. The genome sequences of the three strains, including existing marker sequences such as 16S rDNA genes, were very similar, barring relevant structural variation regions, suggesting that the variation in the dominant *A. hadrus* strain was newly detected using comprehensive scLR sequencing.

## 3. Discussion

Obtaining accurate and complete target bacterial genomes from multiple strains is important for studying the characteristics of uncultured environmental bacterial strains. Although advances in long-read sequencing and associated analysis technologies have made it possible to assemble circular bacterial genomes from cultured strains or metagenomes, obtaining complete bacterial genomes by combining long-read sequencing with uncultured bacterial single-cell MDA products was challenging. One reason for this difficulty was that the single-cell genome was not uniformly amplified during the whole-genome amplification reaction; thus, low-frequency amplified genomic regions were missed from the assembled draft genomes. In this study, we used scALA and obtained circular human gut bacterial SAGs. In particular, we constructed the SAGs by iteration of existing genome assembly algorithms and mapping-based debiasing using scLR sequencing.

Multiple single-cell MDA products derived from the same bacterial strains that exhibit high levels of genome quality are required to construct uncultured bacterial cSAGs using scALA. Because the genome completeness of individual SAGs rarely reached 100% and most SAGs had a genome completeness of 40–60%, long-read sequencing of pooled samples of multiple single-cell MDA products is required to cover whole genomic information. Moreover, chimeric sequences randomly generated in each single-cell MDA product can be detected and removed from pooled sequence data as shown in our previous study (Kogawa et al., 2018). Integration of sufficient SAG data ensures the acquisition of high-quality composite genomes from single-cell MDA products. However, genome completeness and amplification bias exhibited by each SAG in short-read sequencing results should be considered when pooling SAGs and determining the mixing ratio to reduce MDA-derived sequencing bias efficiently. So far, acquiring multiple qualified specific-strain SAGs is challenging owing to low genome quality, high running costs, and the complexity of experimental operations in conventional single-cell sequencing. In this study, we used cost-effective and high-throughput single-cell sequencing SAG-gel platform (Chijiiwa et al., 2020; Arikawa et al., 2021; Hosokawa et al., 2022) to easily obtain hundreds of SR-SAGs and multiple LR-SAGs of specific gut bacterial strains with the required quality for scALA.

Comparative genomics of *A. hadrus*, *A. rectalis*, and *R. gnavus* cSAGs revealed strain-unique structural variations, whereas they showed high homology in the aligned genomic regions. The strain-specific structural variations could be challenging to validate with conventional metagenomic approaches, including 16S rRNA gene amplicon sequencing and mapping short-read metagenomic sequencing reads to known reference genomes. Structural variations can be critical in determining the phenotype of individual bacterial strains, including the presence or absence of the gene sets such as sugar metabolism system, CRISPR-Cas system, or a synthetic flagellar module (Supplementary Table S6). Moreover, our results indicated that analyzing CRISPR-Cas systems could reveal a more accurate evolution and distribution of *A. hadrus* than conventional marker gene analysis could. Metagenomic long-read sequencing used to construct circular bacterial genomes has been reported recently; however, the conventional accuracy of long-read sequences and assembly algorithms often render binning metagenomic sequences from the closely related bacterial species challenging (Moss et al., 2020). Therefore, obtaining circular genomes of specifically targeted gut bacterial species using single-cell genome sequencing will likely be helpful in characterizing bacteria and detecting specific structural variations and gene sets in their genomes.

*Anaerostipes hadrus*, whose genome was obtained in this study, is a notable host-health-associated gut bacteria candidate (Zeevi et al., 2019). Numerous studies on gut bacteria have been conducted, and the gut bacterial genome database is expanding (Almeida et al., 2021). These studies have also suggested the importance of investigating both representative genome sequences of specific species and geographically specific genome features. In this study, we confirmed that sequence similarity of *A. hadrus* genomes did not necessarily correlate with the presence of orthologous functional genes, whereas host geographical regionality appeared to be highly related to gene possession. In contrast, the *A. rectalis* genome highly correlated with sequence similarity, gene possession, and geographical location of the host, which is consistent with a large-scale comparative analysis with more than 1,300 *A. rectalis* genomes (Karcher et al., 2020; Supplementary Figure S5). Our results suggested that the accumulation of type-strain genomes per geographical region was essential for the functional prediction of gut bacteria, and that suitable geographical resolution of strain classification depended on bacterial species.

The *A. hadrus* G001c10 genome contained a mixture of sequences that were in the genomes of two strains collected 9 months earlier, the strain G011c23 from the same host and strain G010c01 from the cohabitant. The position of structural variations and SNPs indicated that the G001c10 genome had a partially chimeric genome consisting of the G010c01 and G011c23 genomes (Figure 5A). Therefore, the emergence of strain G001c10 might be caused by homologous recombination of the G010c01 and G011c23 genomes. Additionally, *A. rectalis* G004c08 and *R. gnavus* G008c02 genomes were obtained after 9 months; however, their structures hardly changed (data not shown). These results indicated that structural variation in the *A. hadrus* genome occurred at a high frequency compared with that in other gut bacteria, resulting in a loss of correlation between sequence homology and gene patterns.

Single-cell genome sequencing typically presents two primary challenges stemming from whole-genome amplification: chimeric sequences (Bankevich et al., 2012) and amplification bias (Nishikawa et al., 2015). Accordingly, we developed scALA for *de novo* assembly of scLR sequences into circular SAGs while addressing these issues. ScLR sequencing with scALA can be utilized to obtain complete circular genomes of uncultured bacteria in humans, soil, marine, and other polar environments, enabling assessment of genomic structural variations. Such structural variations, such as inversions of promoter regions and gene sequences, can alter gene expression and phenotypes (Guérillot et al., 2019). Moreover, the acquisition of complete genomes facilitates the discovery of full-length prophage sequences and the elucidation of bacteria-phage interactions (Marbouty et al., 2021). Our results suggest that strain-specific genomic regions arose from multiple host-specific phage infections in the past. Furthermore, our study highlights the significance of continuously accumulating strain-resolved bacterial genomes to identify gene module alterations within species and understand their relationship with bacterial metabolic traits. Our method expands the capacity for uncultured strain genome comparison to identify novel genes and structural variations, which is made difficult through known reference genome-based metagenomic analyses (Zeevi et al., 2019). The technique we developed can identify unknown bacterial genomes using the closed genomes of single uncultured bacterial cells and deepen our comprehension of intra-species diversity.

## 4. Methods

### 4.1. Experimental design and sample collection

The participants signed written informed consent, and the Ethics Review Committee of the Waseda University has approved the study (No. 2018–323). All methods used were conducted in accordance with the guidelines and regulations outlined by the ethics committee. The participants collected fresh feces into 15-mL vials containing 3 ml of guanidine thiocyanate (GuSCN) solution (TechnoSuruga Laboratory Co., Ltd.), and the samples were stored at 25°C for a maximum of 2 days prior to single-cell encapsulation in droplets.

*Escherichia coli* strain K-12 (ATCC 10798, genome size: 4.6 Mbp) was used for model analysis of cultured cells. *E. coli* K-12 cells were pre-cultured in Luria-Bertani medium for 16 h and collected using centrifugation. The collected cells were washed thrice with ultraviolet-treated Dulbecco’s Phosphate-Buffered Saline (DPBS, Thermo Fisher Scientific).

### 4.2. Single-cell genome sequencing using single-amplified genome-gel platform

Single-cell genome amplification was performed using the SAG-gel platform, according to our previous report (Chijiiwa et al., 2020; Nishikawa et al., 2022). For gut bacteria analysis, after homogenization of human feces in phosphate-buffered saline (PBS) or GuSCN solution (500 μl), the supernatant was recovered by centrifugation at 2,000 × g for 30 s, followed by filtration through a 35-μm nylon mesh and centrifugation at 8,000 × g for 5 min. The resulting cell pellets were resuspended in PBS and centrifuged twice at 8,000 × g for 5 min.

Prior to single-cell encapsulation, cell suspensions were adjusted to 0.1 cells/droplets in 1.5% agarose in PBS to prevent encapsulation of multiple cells in single droplets. Using an On-chip Droplet Generator (On-chip Biotechnologies Co., Ltd), single bacterial cells were encapsulated in droplets floating in a carrier oil, 2% Pico-Surf 1 in Novec 7,500 (Dolomite Microfluidics) and were collected in a 1.5-mL tube, which was chilled on ice for 15 min to form the gel matrix. Following solidification, the collected droplets were broken with 1H,1H,2H,2H-perfluoro-1-octanol (Sigma-Aldrich) to collect the capsules. The gel capsules were washed with 500 μl of acetone (FUJIFILM Wako Pure Chemical Corporation), and the solution was mixed vigorously and centrifuged at 2,000 × g for 10 s. The acetone supernatant was removed, 500 μl of isopropanol (FUJIFILM Wako Pure Chemical Corporation) was added, and the solution was once again mixed vigorously and centrifuged at 2,000 × g for 10 s. The isopropanol supernatant was then removed, and the gel capsules were washed thrice with 500 μl of DPBS.

Thereafter, individual cells in beads were lysed by submerging the gel beads in lysis solutions: first solution, 50 U/μL Ready-Lyse Lysozyme Solution (Lucigen), 2 U/ml Zymolyase (Zymo Research Corporation), 22 U/mL lysostaphin (Sigma-Aldrich), and 250 U/ml mutanolysin (Sigma-Aldrich) in DPBS at 37°C overnight; second solution, 0.5 mg/ml achromopeptidase (FUJIFILM Wako Pure Chemical Corporation) in DPBS at 37°C for 6–8 h; and third solution, 1 mg/ml Proteinase K (Promega Corporation) in 0.5% SDS in DPBS at 40°C overnight. The lysis steps were skipped in *E. coli* experiment. At each reagent replacement step, the gel beads were washed thrice with DPBS and then resuspended in the next solution. After the lysis, the gel beads were washed with DPBS five times, and the supernatant was removed. Thereafter, the beads were suspended in Buffer D2 (denaturation buffer) and subjected to MDA reaction using a REPLI-g Single Cell Kit (QIAGEN).

After MDA at 30°C for 2 h and 65°C for 3 min, the gel beads were washed thrice with 500 μl of DPBS. The beads were then stained with 1× SYBR Green (Thermo Fisher Scientific) in DPBS. After confirming DNA amplification based on the presence of green fluorescence in the gel, fluorescence-positive beads were sorted into 0.8 μl of DPBS in 96-well plates using a BD FACSMelody cell sorter (BD Bioscience) equipped with a 488-nm excitation laser. After droplet sorting, 96-well plates proceeded through the second-round MDA or were stored at ˗30°C.

Following gel bead collection in 96-well plates, second-round MDA was performed using the REPLI-g Single Cell Kit. Buffer D2 (0.6 μL) was added to each well and incubated at 65°C for 10 min. Thereafter, 8.6 μl of MDA mixture was added and incubated at 30°C for 120 min. MDA reaction was terminated by heating at 65°C for 3 min. After second-round amplification, master library plates of single-cell MDA products were prepared. For quality control, single-cell MDA product aliquots were transferred to replica plates, which were used for DNA yield quantification using a Qubit dsDNA High Sensitivity Assay Kit (Thermo Fisher Scientific).

### 4.3. Short-read sequencing

For sequencing analysis, scSR libraries were prepared from the second-round MDA product using QIAseq FX DNA Library Kit (QIAGEN). The ligation adapters were modified to TruSeq–Compatible Full-length Adapters UDI (Integrated DNA Technologies, Inc). Each SAG library was sequenced using an Illumina HiSeq 2× 150 bp configuration (Macrogen).

Furthermore, scSRs quality-controlled with fastp 0.20.0 (Chen et al., 2018b; option: -q 25-r -x) were assembled to SR-SAG *de novo* using SPAdes 3.14.0 (options for SAG: --sc --careful --disable-rr --disable-gzip-output -t 4 -m 32), and contigs of less than 1,000 bp were excluded from subsequent analyses (Bankevich et al., 2012). SR-SAGs with the completeness of >10% and contamination of <10% were selected using CheckM v1.1.2 (Parks et al., 2015). ANI was calculated for the selected SR-SAGs using FastANI 1.3 (Jain et al., 2018). The homology of common single-copy marker genes obtained using CheckM taxonomy workflow (option: --nt --tab_table -t 16 domain Bacteria) was calculated using blastn 2.9.0+ (Camacho et al., 2009) with default options. From individual host datasets, SAGs with ANI of >95%, single-copy marker gene homology of >99%, and tetra-nucleotide frequencies correlation (Teeling et al., 2004) of >90% were classified in the same strain group. The SR-SAGs in strain groups were integrated into CSR-SAGs using ccSAG for chimera removal and co-assembly (Kogawa et al., 2018). Unless otherwise stated, the analysis tools were run with default parameters.

### 4.4. Long-read sequencing and standard *de novo* assembly

We prepared scLR libraries from individual single-cell MDA products or pooled multiple single-cell MDA products using the Rapid Sequencing Kit (Oxford Nanopore Technologies) and sequenced them with Flow Cell R9.4 using a GridION (Oxford Nanopore Technologies). In the case of *E. coli* model sample, scLRs (300 Mbp) were obtained from five *E. coli* second-round MDA products and then integrated into a single file.

In the case of the fecal bacterial sample, we selected second-round MDA products of fecal bacteria from 96-well plates based on strains identified using CSR-SAGs and pooled them (2–8 SAGs per strain) as a single sequencing library for scLR sequencing using a nanopore sequencer.

For *de novo* assembly, we used miniasm 0.3 (Li, 2016; using paf file output by minimap2 2.17 (Li, 2018) with “-x ava-ont” option) or, Canu 1.9 (Koren et al., 2017; -nanopore-raw), and Flye 2.7 (Kolmogorov et al., 2019; --nanopore-raw) with 4.6 Mb genome size option for the assembly of scLRs into LR-SAGs. Miniasm and Flye assembly of the chimera-removed long read was conducted with “trimmedReads” file output by Canu. LR-SAG quality was assessed using QUAST 5.0 (Gurevich et al., 2013) and CheckM (the same option as above). The alignment results of the draft genome and reference *E. coli* genome were visualized using D-GENIES 1.2.0 (Cabanettes and Klopp, 2018). Unless otherwise stated, the analysis tools were run with default parameters.

### 4.5. LR-SAG assembly using single-cell amplified genome long-read assembly

We removed low-quality scLRs of <1,000 bp or with an average quality score of <10 after removing the first 75 bases from the input scLRs using NanoFilt (De Coster et al., 2018; options: “-q 10 -l 1000 --headcrop 75)”. We then constructed intermediate contigs as references for scLR debiasing by *de novo* assembly of the input scLRs. All assemblies in scALA were performed by chimeric sequence trimming using Canu 1.9 (Koren et al., 2017) followed by *de novo* assembly of the scLRs using Flye 2.7 (Kolmogorov et al., 2019). For assembly, chimeric removal was performed by Canu with “-nanopore-raw saveReads = true stopAfter = trimming,” and Flye assembly of the “trimmedReads” file was performed with the “--nano-corr” option. The genome size options of both assemblers were specified with the total length of the CSR-SAGs of the same bacterial strains. Debiasing of the scLRs was then performed. The input scLRs were mapped against the assembled intermediate reference contigs using minimap2 2.17 (Li, 2018). Biased sequences were identified according to the mapped depth, and the excess number of the input scLRs was removed to be 50× sequence depth using biostar154220.jar of JVarkit (Lindenbaum, 2015). Even if the average depth was less than 50×, read subsampling was performed for the areas that exceeded 50× depth. By re-assembling using the bias-reduced scLRs, we constructed the second cycle reference contigs, which cover a more comprehensive range of genomic regions than do the first reference contigs, and proceeded to the next debiasing step. If the total length of the reassembled contig did not change, the assembly and debiasing cycle was stopped, and we repeated the assembly and debiasing processes four times in this study. Consensus sequences were then constructed by the scaffolding of the representative intermediate reference contig with the smallest number of contigs. For the scaffolding, the BLAST alignment result of the representative reference contig to other reference contigs and Multi-CSAR (Chen et al., 2018a) was used. After the scaffolding, the sequences were polished using Pilon 1.22 (Walker et al., 2014) with scSRs obtained from the same MDA product. After polishing the sequences, we obtained strain-specific LR-SAGs. In the case of fecal bacterial genome sequencing, closed cSAGs of *A. hadrus*, *R. gnavus*, and *A. rectalis* were constructed by gap-filling LR-SAG contigs with SR-SAGs of the same single-cell MDA products. The SR-SAGs were aligned to LR-SAGs by the blast and the “N”s of the gap region covered by the SR-SAG contigs were replaced. Unless otherwise noted, the analysis tools were run with default parameters.

### 4.6. Comparative genome analysis

Pan-genome analysis was performed using Roary (Page et al., 2015; options: -n -e) with reference genomes in the NCBI genome database. Clustering of strains was performed using UMAP analysis based on the presence of homologous gene groups (Emms and Kelly, 2019). Distance matrices of the genomes were generated using the Manhattan method and visualized using the R package umap 0.2.7 (McInnes et al., 2018) with the “n_neighbors” option corresponding to each figure. For phylogenetic analysis, SNP-based trees were generated by the maximum likelihood method using RAxML-NG 0.9.0 (Kozlov et al., 2019; options: --model GTR) from the core gene alignment result of MAFFT 7.245 (Katoh and Standley, 2013) in the Roary analysis process. The tree was illustrated using iTOL 6.4.3 (Letunic and Bork, 2021).

Genome alignment results of LR-SAGs were visualized using D-GENIES1.2.0 (Cabanettes and Klopp, 2018) or Circos 0.69 (Krzywinski et al., 2009) for bacterial genomic structural variation analysis. In addition, KEGG annotation using KofamScan (Aramaki et al., 2020) and VOG annotation using VIBRANT 1.2.1 (Kieft et al., 2020) were performed on coding sequences (CDSs) extracted using Prokka 1.14.5 (Seemann, 2014; options: --compliant). The t-test of VOG gene possession was performed with R. Gene modules were plotted with genoPlotR (Guy et al., 2010). Pathway analysis and CRISPR detection were performed using DRAM 1.0.6 (Shaffer et al., 2020) and CRISPRDetect 2.4 (Biswas et al., 2016), respectively. The presence of carbohydrate-active enzymes (CAZy) is visualized from the DRAM analysis result. Unless otherwise noted, the analysis tools were run with default parameters.

## Data availability statement

The datasets presented in this study can be found in online repositories. The names of the repository/repositories and accession number(s) can be found at: https://www.ncbi.nlm.nih.gov/, PRJNA818799 and PRJNA692334. The scALA pipeline is available on Github (https://github.com/mstkgw/scALA).

## Ethics statement

The studies involving human participants were reviewed and approved by the Ethics Review Committee at Waseda University. Written informed consent to participate in this study was provided by the participants’ legal guardian/next of kin.

## Author contributions

MK and MH conceived and designed the experiments, analyzed all data, and wrote the original manuscript. MK, YN, HT, and MH developed the long-read single-cell sequencing. MK, TS, and TY conducted the genomics experiments and collected the data. MK and KA conducted bioinformatics analysis of single-cell genomic data. All authors reviewed and approved the final manuscript.

## Funding

This work was partly supported by JST-PRESTO (grant number JPMJPR15FA) and MEXT KAKENHI (grant numbers 21H01733 and 19 K22089).

## Conflict of interest

MH and HT are shareholders in bitBiome, Inc., which provides single-cell genomics services using the SAG-gel workflow as bit-MAP. MH is a founder of bitBiome, Inc. MK, TS, TY, and KA are employed at bitBiome, Inc. MK, MH, YN, KA, and HT are inventors on patent applications submitted by bitBiome, Inc., covering the technique for single-cell sequencing.

## Publisher’s note

All claims expressed in this article are solely those of the authors and do not necessarily represent those of their affiliated organizations, or those of the publisher, the editors and the reviewers. Any product that may be evaluated in this article, or claim that may be made by its manufacturer, is not guaranteed or endorsed by the publisher.
